# Improving attachment style clustering with ROCKET and CatBoost: Insights from EEG analysis

**DOI:** 10.1371/journal.pone.0331112

**Published:** 2025-09-02

**Authors:** Dor Mizrahi, Ilan Laufer, Inon Zuckerman

**Affiliations:** Department of Industrial Engineering and Management, Ariel University, Ariel, Israel; University of Exeter, UNITED KINGDOM OF GREAT BRITAIN AND NORTHERN IRELAND

## Abstract

Understanding attachment styles is essential in psychology and neuroscience, yet predicting them using objective neural data remains challenging. This study explores the use of machine learning (ML) models and EEG analysis to improve attachment style classification. We analyzed EEG data from 27 university students (ages 20–35) with attachment styles categorized as secure, avoidant, anxious, or fearful-avoidant, assessed using the ECR-R questionnaire. EEG features were extracted using the ROCKET algorithm, followed by Principal Component Analysis (PCA) for dimensionality reduction. The CatBoost algorithm was used for prediction, with a two-stage data pruning approach to enhance accuracy. Our model showed a strong relationship between the number of EEG epochs and predictive accuracy, with Secure and Fearful-Avoidant attachment styles being predicted most reliably. Anxious and Avoidant styles exhibited greater variability, reflecting their complex neural signatures. These findings support the idea that attachment exists on a spectrum rather than as fixed categories, influenced by life experiences, emotional regulation, and social context. The results reinforce the dimensional nature of attachment and highlight the trade-off between model accuracy and computational efficiency. This study demonstrates the potential of ML-driven EEG analysis in predicting attachment styles, offering new possibilities for psychological assessment. By identifying overlapping neural signatures, our findings highlight attachment as a dynamic rather than static process, which could inform clinical interventions and future research on neural markers of attachment.

## 1. Introduction

Attachment theory, established by John Bowlby [1,2], is a fundamental framework for understanding the emotional and behavioral dynamics in human relationships. The constructs of anxiety and avoidance are critical within this framework, shaping our comprehension and evaluation of interpersonal relationships and individual psychological states [1–3]. Recent EEG research has identified neurophysiological markers linked to these attachment dimensions [4–7]. However, despite these advancements, the development of precise AI predictive models from EEG data is an emerging field, with studies in this area being scarce. This situation offers a valuable opportunity for new research contributions.

This study aims to demonstrate that through data refinement, AI can be leveraged to predict individual attachment styles from EEG data with enhanced accuracy. We advance upon our prior work, which primarily classified attachment into secure and insecure categories [3,4], by adopting a more nuanced approach. To this end, we have incorporated the CatBoost algorithm, renowned for its proficiency with categorical data and regression tasks [5,6], to predict each of the four established attachment styles—secure, avoidant, anxious, and fearful-avoidant [7]. The novel contribution of our research is twofold. Methodologically, we incorporate the ROCKET [8,9] algorithm to extract a comprehensive feature set from the EEG data, capturing temporal and spatial patterns that hold significance for attachment-related behaviors. To manage the complexity of the features extracted by ROCKET, we utilize Principal Component Analysis (PCA) to distil the data into its most informative components to ensure the clarity and relevance of our model’s inputs.

Recognizing the importance of dataset integrity in machine learning [10], particularly for the multifaceted EEG data pertinent to psychological constructs, we have instituted a robust data pruning (e.g., [11]) methodology. Our bespoke pruning process systematically sifts through EEG epochs, employing a data-centric approach to select the most representative segments of the EEG recordings. This selection is guided by a custom algorithm that evaluates the proximity of each epoch’s attachment values to the central tendency of the dataset, iteratively refining the pool of data to enhance the model’s predictive precision. By leveraging the advanced feature extraction capabilities of the ROCKET algorithm alongside the regression proficiency of the CatBoost algorithm, our study introduces a two-stage predictive model designed to more accurately capture the subtleties of attachment styles reflected in EEG data. The anticipated outcomes of this research may open avenues for more precise psychological assessments and illustrate the significant promise of interdisciplinary approaches in the field of psychological AI research.

## 2. Methods

In this section, we outline the methodology employed in our study to predict attachment styles from EEG data. We describe the data collection process, feature generation and dimensionality reduction techniques, the predictive modeling algorithm, and our approach to iterative refinement and data efficiency. Additionally, we explain how we assessed the model’s performance across different scenarios, including attachment classes and values of N (number of epochs), and discuss the use of permutation testing to ensure the robustness of our results.

### 2.1. Ethical statement

The experimental protocols employed in this study were reviewed and approved by the Ethics Committee of Ariel University (approval number: AU-ENG-IZ-20220404). All experiments were conducted in accordance with relevant guidelines and regulations. Permission for electrophysiological recordings was granted for the period from February 4, 2022, to February 4, 2023. Written informed consent was obtained from all participants prior to their inclusion in the study.

### 2.2. Participant recruitment period and process

The recruitment process for this study consisted of two stages. In the first stage, from **April 10, 2022** to **April 20, 2022**, **103 subjects** completed the ECR-R [12,13] attachment questionnaires distributed to all volunteers. Based on their responses, **27 participants** were selected for the second stage, which involved an EEG session in the laboratory. The EEG data collection took place over multiple days, beginning on **May 17, 2022** and concluding on **June 1, 2022**.

### 2.3. EEG data collection and epoch extraction

In our investigation into the varying patterns of attachment, we employed a sequential, dual-phase methodology. Initially, we curated a sample of 96 candidates, sourced from the senior-year cohort of an engineering program, aged 20–35 years (mean age = 24.25, standard deviation = 2.0673). Criteria for selection included right-handedness and the absence of any neurological concerns. The attachment typologies for these individuals were determined using the ECR-R questionnaire, a recognized tool in psychological assessments [12,13]. Following this, a k-means clustering algorithm stratified the sample into four attachment typologies—secure, anxious, avoidant, and fearful-avoidant, as delineated in recent research [14–16].

For the EEG phase that followed, 27 subjects were chosen to ensure balanced representation from both the secure and the diverse insecure attachment groups, adhering to proportional quotas reflective of the initial clustering outcomes. The secure group consisted of 6 participants, while the insecure group included 21 participants (9 anxiously attached, 7 avoidant, and 5 fearful-avoidant). Detailed EEG procedural nuances and participant distribution specifics are documented in our recent publication [3].

**Fig 1:**
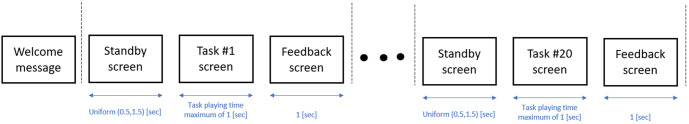
Experimental paradigm – single block.

During EEG monitoring, participants performed a cognitive task known as the flanker task, as detailed in [17]. The task consisted of 60 stimuli, organized into three sets of 20 (see Fig 1). In the middle set, participants were instructed to oppose their responses to the directional cues of arrows, while for the other two sets, they aligned their responses with the cues. One-minute intermissions separated these sets. Performance feedback was instantaneous, marked by a green or red signal, with an exposure of one second. The trials were interspaced with a neutral visual prompt, lasting between half a second to one and a half seconds, prepping the participant for the next stimulus. Each complete set spanned an approximate duration of one minute. An orientation session prefaced the main task to familiarize subjects with the expected actions. In total, we had 1600 epochs, 1240 samples of the insecure classes (anxious, avoidant, and fearful-avoidant) and 360 samples of the secure class.

**Fig 2:**
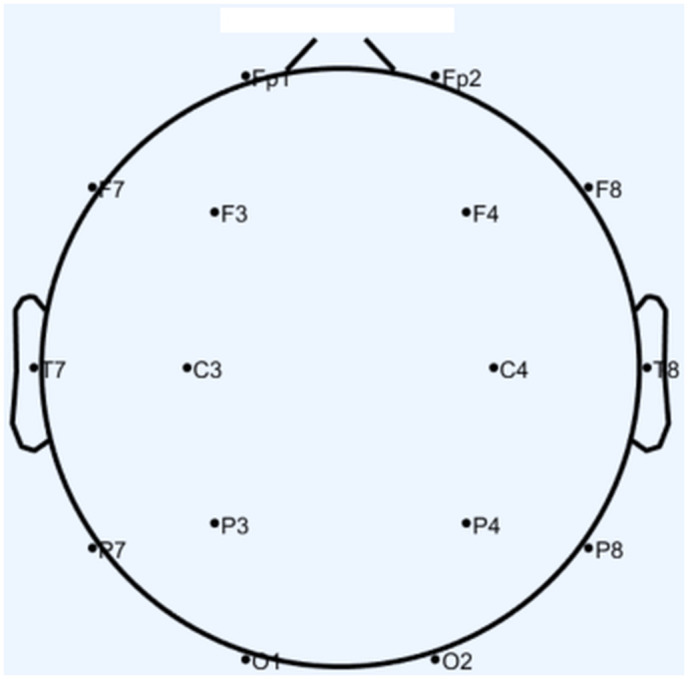
Schematic representation of the 16-electrode montage used in this study, following the standard 10–20 EEG system.

In capturing the EEG data, we utilized a 16-channel EEG apparatus (USBAMP, g.tec, Austria), calibrated for a sampling frequency of 512 Hz and congruent with the 10–20 international positioning framework (see electrodes layout in Fig 2). We concentrated on monitoring the frontal and prefrontal cortical areas, specifically targeting six electrode sites: Fp1, F7, Fp2, F8, F3, and F4. To preserve key neural frequencies associated with cognitive and affective processing while minimizing low-frequency drift and high-frequency artifacts, we applied a bandpass filter set to 1–30 Hz. This range adequately captures alpha (8–12 Hz) and low-beta (13–30 Hz) activity, which prior EEG research has linked to attentional, emotional, and executive processes [18,19]. Following this, an Independent Component Analysis (ICA) was conducted to further segregate genuine neural activity from ocular and muscle artifacts, ensuring the resulting signals are more representative of true cortical processes. After preprocessing, the continuous data were chronologically segmented into one-second epochs, each aligned with the visual cues of the flanker task, thereby allowing for a more precise analysis of moment-to-moment fluctuations in neural activity.

### 2.4. Feature generation and dimensionality reduction

In our exploration of EEG data for attachment style prediction, we employed the ROCKET algorithm [8], utilizing its default configuration of 10,000 kernels to initially generate a rich feature set of 20,000. Although further kernel-level tuning is possible, we found the standard configuration sufficient, given that our subsequent PCA step retained over 90% of the variance in just 87 principal components, effectively mitigating the need for additional parameter adjustments. We selected ROCKET due to its proven effectiveness in time-series classification, particularly with high-dimensional EEG data, and its ability to capture a broad range of temporal features via randomized convolutional kernels [8,20]. The kernels applied in ROCKET are random and convolutional, allowing them to capture diverse temporal patterns in the EEG signals, including frequency content, transient events, signal complexity, and amplitude variations that are relevant to understanding underlying neural activity. However, because this large number of features exceeds our total number of observations, it raises a high risk of overfitting and complicates the subsequent analysis. To streamline this extensive collection of features and facilitate a more efficient analysis, Principal Component Analysis (PCA) was subsequently applied. This dimensionality reduction process refined the initial feature set down to 87 principal components while preserving over 90% of the variance, thus providing a more efficient input space for our predictive models and balancing the richness of the extracted features with the need for computational tractability and robust model performance.

### 2.5. Predictive modeling and algorithm

Our predictive framework utilizes the CatBoost algorithm [5], adeptly tailored for dual regression tasks, allowing for the simultaneous prediction of the attachment dimensions ‘avoidance’ and ‘anxiety.’ We selected CatBoost for its robust performance in multi-target regression tasks and its ability to effectively handle correlated label dimensions, making it particularly suited for predicting both ‘avoidance’ and ‘anxiety’ simultaneously (e.g., [21]). To achieve the lowest validation loss, we performed a grid search over hyperparameters including “learning_rate”, “max_depth”, “l2_leaf_reg”, “bagging_temperature”, and “random_strength”, ultimately settling on learning_rate = 0.03, max_depth = 6, l2_leaf_reg = 3, bagging_temperature = 1, and random_strength = 1. Optimization of predictive accuracy was achieved through an RMSE cost function, adept at minimizing spatial distances in the label space and improving the precision of our attachment style predictions.

To fully utilize the dataset and reduce the risk of overfitting, we trained the model using the K-fold cross-validation (K = 3) method [22]. This approach ensured that all data samples contributed to both training and validation at different stages, providing a more generalized evaluation. Importantly, all reported results were obtained from instances where the relevant sample was in the test fold, ensuring unbiased performance assessment.

When using CatBoost for this dual regression task, the following adaptations and considerations were made:

a **Dual Regression Modeling:** We employed CatBoost’s regression capabilities [6] to perform regression analysis for predicting attachment styles, with avoidance and anxiety serving as the two primary dimensions. This allowed us to model and predict attachment styles as continuous variables, providing insights into the detailed variations of these dimensions within our dataset. The algorithm has been specifically configured and fine-tuned by searching for the best combination of hyperparameters to effectively handle the simultaneous prediction of two continuous variables that are likely correlated with each other.
b **Cost Function Customization**: In our study, we harnessed the customization capabilities of CatBoost [6,23] to tailor the cost function to our specific needs. Notably, we chose to use multi-RMSE (Root Mean Square Error) as our primary cost function, allowing us to fine-tune the model to minimize the RMSE between predicted and actual values. This customization played a crucial role in shaping the model’s training and refinement, aligning it closely with our research objectives. RMSE serves as a widely employed metric in regression tasks, quantifying the disparities between predicted and actual values. In cases involving multiple target variables for prediction, known as multi-target regression problems, the multi-RMSE is employed as an assessment measure [24]. The multi-RMSE cost function we used is a modification that accounts for the relationship between ‘avoidance’ and ‘anxiety’ (see Equation 1), minimizing errors across both dimensions simultaneously rather than treating them as separate, unrelated predictions.

$$Multi\;RMSE=\;\sqrt{\frac{1}{n}\sum_{i=1}^{n}\left(\frac{1}{d}\sum_{j=1}^{d}{\left(y_{ij}-{\hat{y}}_{ij}\right)}^{2}\right)}\;$$ (1)

Where:

-n is the number of observations (epochs in the context of EEG)

-d is the number of dimensions (attachment dimensions in this case)

-$y_{ij}$ is the true value of the i^th^ observation and j^th^ dimension

-${\hat{y}}_{ij}$ is the predicted value of the i^th^ observation and j^th^ dimension

### 2.6. Iterative refinement and data efficiency

An innovative scheme was applied to refine the EEG epoch data set further, focusing on the most representative epochs by averaging attachment values and selecting those closest to the predictive target. This iterative process aimed to filter out high-variance epochs and concentrate on data that most accurately represented participant attachment styles.

To enhance the accuracy of attachment style prediction using EEG data, we developed a bespoke two-stage algorithm inspired by the core steps in the k-means clustering procedure [25] rather than applying standard k-means clustering. Specifically, our algorithm mirrors k-means in its iterative “distance measurement” and “centroid (mean) update” steps, but it operates on a single evolving mean attachment value (instead of multiple centroids) and prunes outlier epochs at each iteration. As such, we do not perform clustering in the traditional sense; instead, we iteratively refine the dataset to emphasize the most representative epochs.

Here’s a detailed breakdown of our algorithm, assuming we have N EEG epochs

**Stage One: Initial Attachment Value Estimation**

a **Value Calculation for Each Epoch**: For each of the N EEG epochs, we calculate a two-dimensional attachment value, representing ‘anxiety’ and ‘avoidance’.
b **Mean Attachment Value Computation**: We integrate the individual attachment values from all epochs to form an aggregated mean. This mean serves as a provisional centroid akin to the k-means process, representing a collective attachment tendency.

**Stage Two: Refinement and Iterative Process**

c **Distance Measurement**: The Euclidean distance from each epoch’s attachment value to the mean attachment value is calculated.
d **Selection of Representative Epochs**: We refine our epoch set by retaining only the subset comprising the closest half (N/2) of epochs, effectively reducing noise and high-variance outliers.
e **Recalculation of Mean Attachment Value**: With the pruned subset, we compute a new average attachment value, yielding a more precise representation of the underlying attachment styles.

These two stages loosely parallel the assignment and update steps in k-means, but our approach focuses on refining a single evolving mean attachment value rather than partitioning data into multiple clusters.

### 2.7. Evaluating algorithm performance across different N values and attachment classes

In this final step of our evaluation, we assess the algorithm’s performance across a range of increasing values of N, exploring aggregate analysis applied within attachment classes. This comprehensive evaluation yields critical insights into the model’s predictive accuracy and its ability to distinguish between attachment styles. Conducting the analysis within each attachment class individually offers a more detailed examination of the model’s performance for specific attachment styles. This granular approach helps to identify potential variations in predictive accuracy among different attachment classes, shedding light on the model’s strengths and weaknesses in distinguishing between specific attachment profiles.

### 2.8. Assessing robustness with permutation testing

To ensure reliable results for each value of N and participant, we performed 10,000 permutations of epochs for each run of the algorithm. The results were then aggregated for all participants within each attachment class. For example, the secure attachment class consisted of 6 participants, resulting in a total of 60,000 permutations for each value of N (10,000 permutations per participant). This comprehensive approach accounts for all participants within an attachment class, enhancing the model’s performance assessment by reducing the impact of outliers or random variations in individual permutations.

Stage One of our model focuses on establishing a baseline mean attachment value, which serves as an initial approximation. This baseline helps prevent underfitting, where the model might oversimplify and provide inaccurate results. In Stage Two, we undertake an iterative refinement process. During this stage, we systematically assess the robustness of the model by validating its performance across all attachment classes. This iterative refinement, akin to a tuning process, incrementally improves the fidelity of attachment style predictions. By focusing on the most consistent and representative epochs, we minimize the influence of outliers or random variations that could otherwise skew the overall average. This step helps mitigate the risk of overfitting, where the model becomes too specific to the training data and may not generalize well.

### 2.9. Summary of overall methodology

**Fig 3:**
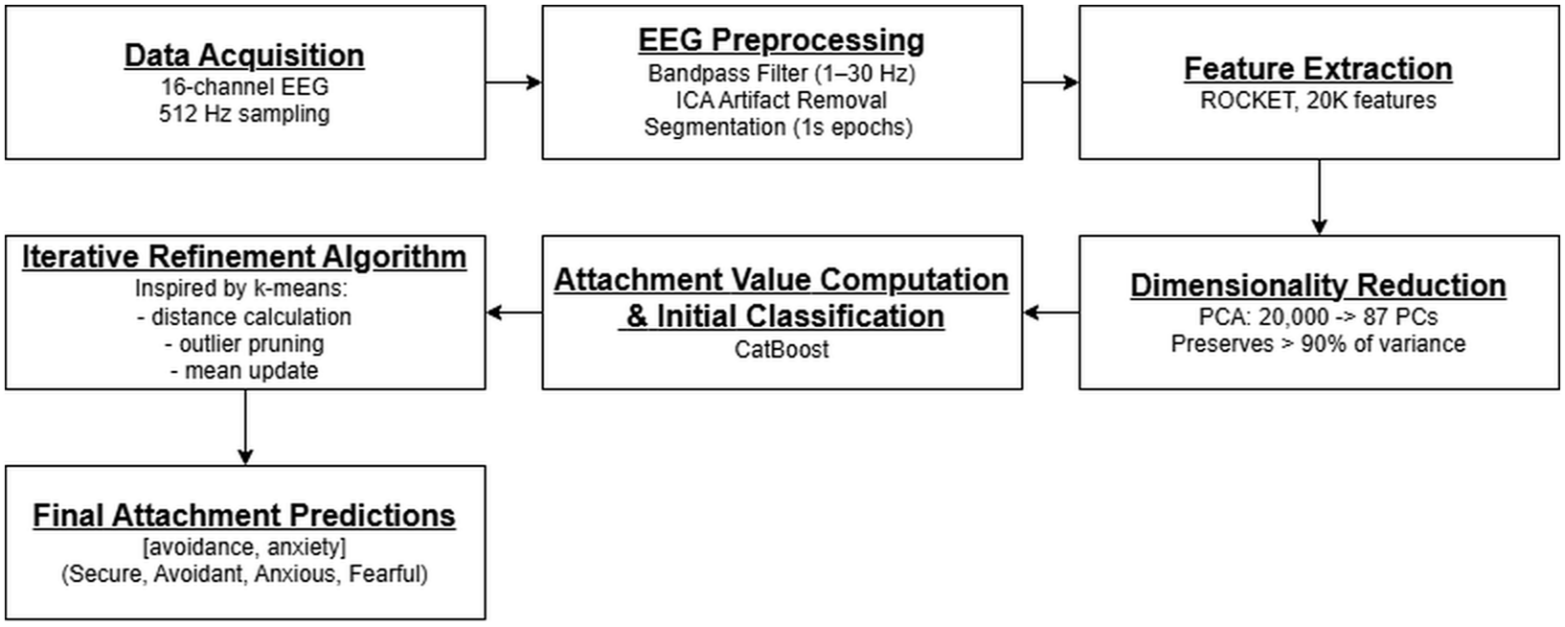
Overall methodological pipeline.

Fig 3 illustrates the integrated workflow of our study, drawing together the procedures described in Sections 2.1–2.7. In brief, we recruited participants and acquired EEG data (Section 2.1–2.2), extracted and refined features using ROCKET and PCA (Section 2.3), applied CatBoost for initial classification (Section 2.4), and further honed predictions using our iterative refinement algorithm (Section 2.5). Finally, we evaluated robustness and performance across different conditions (Sections 2.6–2.7). This holistic perspective demonstrates how each component of our methodology contributes to improved attachment style prediction accuracy.

## 3. Results

In this section, we provide an overview of our study’s results by using a series of figures offering insights into the progressive evolution of our predictive model’s performance. We begin by discussing the model’s initial predictive capabilities and proceed to illustrate how its accuracy improves through selective epoch pruning. Subsequently, we delve into the model’s adaptability across attachment styles and conclude with visualizations of attachment style clusters based on the model’s predictive accuracy. These findings collectively shed light on the dynamics of our model’s development and its enhanced precision in predicting attachment styles. We shall commence with Fig 3.

Fig 3 lays the foundation by elucidating the model’s primary predictive capabilities:

- Ground Truth – Attachment Score (Red Point): This point represents the actual attachment score and acts as a benchmark for assessing the model’s accuracy.
- Single Epoch Predictions (Blue Points): These early predictions, generated after just one epoch, provide an initial glimpse into the alignment of the model with the ground truth.
- Mean Predicted Attachment Score (Green Point): This point represents the average of all predictions across the dataset, summarizing the model’s initial performance.
- Prediction Error (Distance = 0.11577): This numeric value quantifies the initial accuracy of the model by measuring the discrepancy between the predicted and actual score.

**Fig 4:**
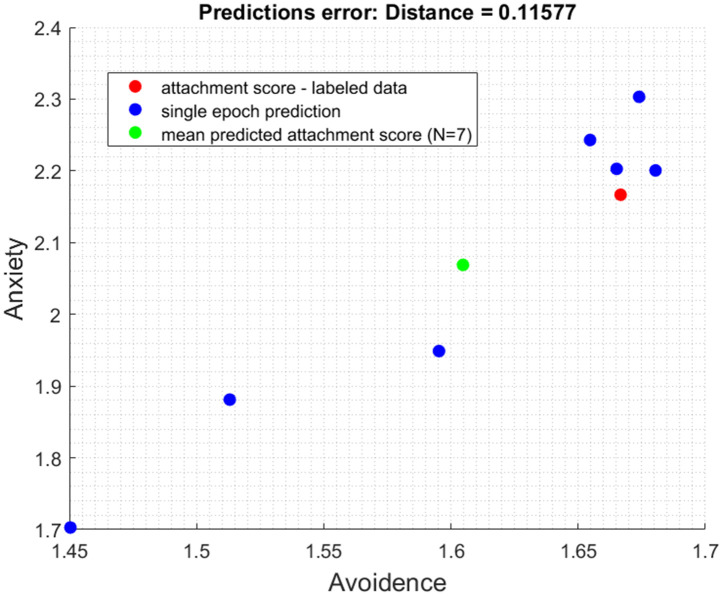
Model’s initial predictive performance (N = 7).

Fig 3 serves as a crucial baseline, offering insights into the model’s initial predictive accuracy and setting the stage for subsequent optimization efforts as depicted by Fig 4.

Fig 4 provides a visual representation of the model’s accuracy improvement through a crucial pruning process:

- Red Point (Ground Truth – Attachment Score): This point represents the actual attachment value of the players as predicted in the questionnaire, serving as the reference.
- Blue Points (Predictions from Selected N/2 Closest Epochs): Each blue point signifies the predictions made by the model using the N/2 epochs closest to the average obtained.
- Magenta Points (Omitted Points): The magenta points indicate the omitted data points that exceeded the distance threshold.
- Green Point (Mean Predicted Attachment Score): This green point denotes the average prediction value obtained by averaging the predictions from all the selected epochs, which are marked in blue.

Fig 4 provides evidence of the model’s ability to enhance its predictive accuracy by discarding less accurate epochs, ultimately reducing the prediction error and bringing predictions closer to the ground truth. The interplay between Figs 2 and 3 offers a visual narrative of the model’s evolution. Comparing Fig 3, which represents the model’s initial, broader predictions, to Fig 4, where the model’s accuracy is enhanced through systematic refinement, we can clearly see a reduction in the distance between the green and red points. This reduction, reflected in the decrease of the prediction error from 0.11577 to 0.07072, signifies a substantial improvement in the model’s quantitative accuracy of 38.91%. It illustrates the algorithm’s progression toward a more detailed understanding of attachment styles.

**Fig 5:**
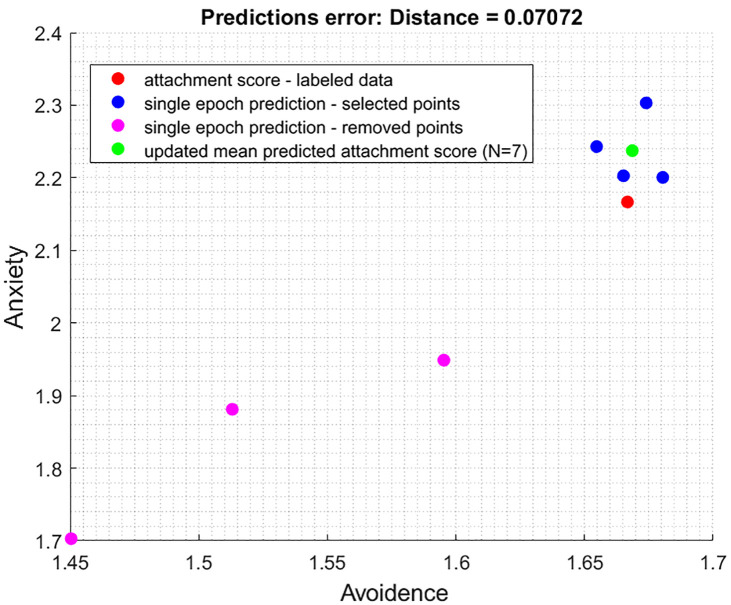
Enhanced model predictions through Epoch selection.

Building upon the foundation established by Figs 2, 3 and Fig 5 below introduces a critical dimension to our understanding of the model’s behavior across different attachment styles through an iterative voting procedure.

Fig 5 offers a detailed exploration of the model’s predictive accuracy enhancement for each attachment style by varying the number of epochs. The figure features attachment style trajectories represented by color-coded lines, distinguishing between Secure (blue), Avoidant (red), Fearful Avoidant (magenta), and Anxiously Attached (green) styles. These lines demonstrate the ‘Mean Distance’ from actual scores as the number of epochs increases from 1 to 39, in increments of two.

To ensure a comprehensive evaluation accounting for group size differences and individual variations, we applied a weighted analysis method across four distinct attachment style groups: Secure (6 participants), Anxiously Attached (9 participants), Avoidant (7 participants), and Fearful Avoidant (5 participants). Within each group, we conducted 10,000 permutations of epoch groups for individual participants, assigning equal weight to every permutation within a participant’s subset. This approach provided an unbiased assessment of each group’s performance, addressing both individual differences and group size disparities while enhancing the validation process’s robustness through various epoch combinations, reducing the influence of outliers and anomalies.

Interpreting the graph’s evolution, it becomes evident that the model’s accuracy improves as it incorporates more epochs into its voting procedure. Specifically, the ‘Mean Distance’ for each attachment style decreases, indicating enhanced accuracy. Additionally, Fig 5 highlights attachment style-specific trends, with differentiated paths suggesting that Secure and Fearful Avoidant styles may require fewer epochs for accurate prediction, while other styles benefit from a larger dataset for improved accuracy.

In summary, Fig 5 is a critical dimension in understanding the model’s behavior across different attachment styles through an iterative voting procedure. It helps elucidate the progression of the model’s accuracy from its initial state (Fig 3) to its refined state (Fig 4) across different N numbers. The figure illustrates how the model enhances its predictive accuracy for each attachment style by varying the number of epochs, with the x-axis representing the number of epochs in the voting procedure. It employs a weighted analysis approach to ensure fairness in evaluating attachment style groups. In the broader context of our findings, Fig 5 serves as a valuable visual representation of the model’s adaptability and accuracy across various attachment styles. It offers insights into the interplay between data volume, predictive reliability, and attachment style variations, showcasing the model’s iterative improvement in predictive performance.

**Fig 6:**
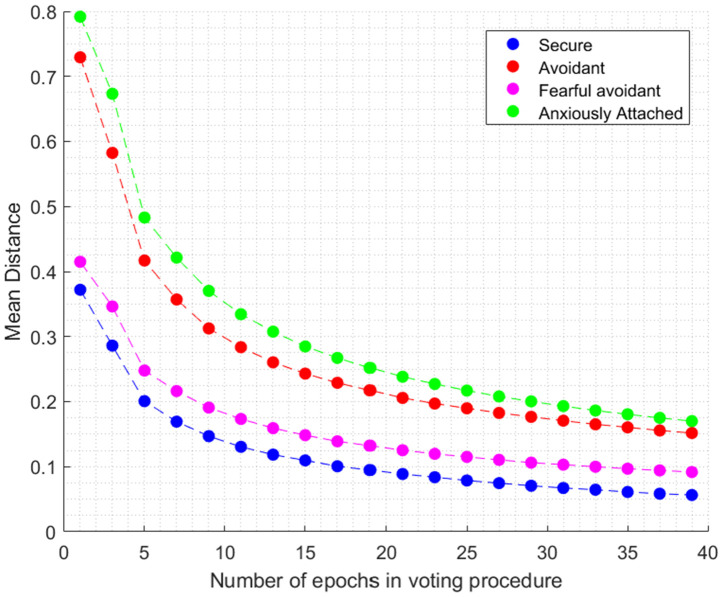
Evolution of predictive accuracy across attachment styles through iterative voting procedure.

Fig 6 presents the average centroid locations calculated using k-means clustering within a two-dimensional space representing Avoidance and Anxiety.

**Fig 7:**
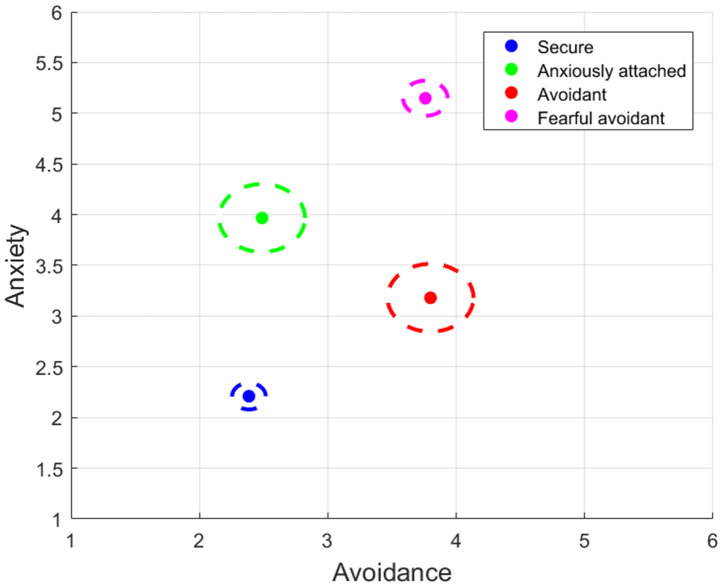
Attachment style clusters at N = 11 – Centroid position and variation spread of individual data points.

The circles in the graph indicate the average distance of data points within each cluster, serving as a proxy for prediction error. The size and compactness of these circles reflect the algorithm’s consistency in classifying each attachment style.

Interpreting the visual information in Fig 6 and considering the radius of each circle, which signifies data compactness or error size, we can draw the following conclusions:

1. Secure Cluster (Blue): The blue circle, representing the Secure attachment style, is tightly clustered with a small radius. This suggests that individuals classified under the Secure attachment style tend to have minimal variability in their Anxiety and Avoidance scores. Consequently, the model’s prediction error for this cluster is expected to be low, indicating high precision in identifying individuals with Secure attachment characteristics.
2. Anxiously Attached Cluster (Green): The green circle has a larger area compared to the blue one, indicating greater variability among individuals within the Anxiously Attached category. This implies that while the central tendency for Anxiously Attached individuals is characterized by higher Anxiety and lower Avoidance, there is less consistency in the model’s predictions for this group, as evident by the larger circle radius.
3. Avoidant Cluster (Red): The red circle is similar in size to the green one, suggesting a comparable level of prediction error or variability for the Avoidant attachment style. Individuals in this cluster exhibit higher Avoidance but lower Anxiety. The spread of this cluster also indicates less consistency in the model’s predictions for this group.
4. Fearful Avoidant Cluster (Magenta): The magenta circle is smaller than the green and red circles but slightly larger than the blue one. This cluster comprises individuals with high levels of both Anxiety and Avoidance. Nevertheless, the relatively tight cluster suggests that the Fearful Avoidant attachment style exhibits predictive accuracy comparable to the Secure group, with relatively less variation in the model’s classification of these individuals compared to those with Anxious or Avoidant attachment styles.

In summary, the Secure cluster stands out with the most compact grouping, emphasizing the model’s exceptional performance in predicting this attachment style. On the other hand, the Anxiously Attached and Avoidant clusters, represented by larger circles, display varying degrees of prediction error and data variability, indicating less consistency in the model’s predictions. The Fearful Avoidant cluster occupies an intermediate position, demonstrating predictive accuracy comparable to the Secure group. In the context of our predictive analysis, Fig 6 showcases the model’s performance when calibrated with N = 11 epochs, a selection based on the observable inflection in the prediction error trend from Fig 5. This demonstrates the algorithm’s ability to cluster different attachment styles with improved accuracy. By choosing N = 11, we strike a pragmatic balance between model accuracy and computational efficiency, making it relevant for real-world applications (see Fig 7.).

## 4. Discussion

Our study endeavors to advance the application of artificial intelligence in psychological assessment by focusing on the accurate prediction of attachment styles through EEG data analysis. To achieve this, we introduce a two-stage approach designed to enhance the precision and efficiency of AI models for predicting attachment styles based on EEG data.

### 4.1. Dual-stage framework for enhanced prediction

The core of our approach centers around a dual-stage framework aimed at improving prediction precision while streamlining computational aspects. In the first stage, we leverage the CatBoost algorithm [5], known for its predictive strength across domains, to establish baseline attachment style predictions using EEG data. This forms the foundation for our subsequent refinement process, which constitutes the second stage of our model. This refinement process involves data pruning [26] (Luo and Wang, 2018) and employs a data sampling scheme within our framework to extract informative neural patterns while adhering to principles of smart sampling [27].

### 4.2. Feature extraction and model analysis

For feature extraction, we utilized ROCKET [8] to process the time-series data, and CatBoost [6] was employed to analyze these features. CatBoost’s proficiency in handling datasets with numerous features was crucial in managing the complexity of attachment style data. We used RMSE (Root Mean Square Error) as a cost function [28] to guide model refinement. The number of epochs was set at 11, as determined by the trend in RMSE observed in Fig 5, balancing model accuracy and computational efficiency. This approach aided in distinguishing between different attachment styles, as demonstrated in Fig 6.

### 4.3. Implications for attachment theory

Our findings hold significance within the context of the dimensional theory of attachment styles [29]. Advanced machine learning techniques allowed us to address the data’s complexity, reflecting the continuous nature of attachment styles. Fig 6 visually underscores the variability in predicting Anxious and Avoidant attachment styles, indicative of broader error ranges in more complex attachment dimensions. These findings support the dimensional perspective of attachment theory, emphasizing that attachment styles span multiple dimensions, enriching our understanding of human attachment and relationships.

Specifically, the Secure cluster exhibits the tightest grouping, indicating the model’s exceptional performance in predicting this attachment style (Fig 6). Conversely, the Anxiously Attached and Avoidant clusters, with larger radii, demonstrate varying prediction errors and data variability, suggesting less consistency and precision. The Fearful Avoidant cluster falls in between, indicating comparable predictive accuracy to the Secure group. These findings harmonize with established literature that posits Secure and Fearful Avoidant attachment occupy opposite ends of the attachment style spectrum [30]. This highlights the complex and continuous nature of attachment styles, supporting the dimensional approach within attachment theory.

### 4.4. Insights for AI researchers

Our study offers insights for AI researchers working with complex, dimensional data. We present a practical example of using ROCKET for time-series data feature extraction, paired with CatBoost for analysis. The use of RMSE as a cost function for model refinement, particularly in determining the number of epochs, demonstrates a systematic approach to model tuning. Indiscriminate increases in the number of epochs may not significantly enhance model performance, introducing unnecessary complexity without gains in accuracy. The error distribution visualizations in our results can serve as a template for transparently displaying prediction challenges in AI research with complex datasets. These two stages, comprising CatBoost-driven baseline predictions and iterative data refinement, form the cornerstone of our approach to improving attachment style prediction through EEG data analysis, with the use of ROCKET for feature extraction.

### 4.5. Broader theoretical implications

Our findings add to the growing understanding that attachment styles exist on a spectrum rather than as fixed categories (e.g., [31,32]). The variation in our model’s predictive accuracy across different attachment styles reflects how attachment is shaped by a mix of psychological and neural factors. This supports the idea that attachment isn’t static, it can shift based on life experiences [33], emotional regulation [34], and social context [35]. By applying EEG-based predictive modeling, we provide further evidence that attachment styles, especially anxious and avoidant patterns, have overlapping and complex neural signatures. This reinforces the notion that attachment is a dynamic process rather than a simple label.

### 4.6. Limitations and future directions

1. Limited Sample Size: Our study’s sample size, especially within the secure attachment group, was limited due to the proportional allocation method used. To ensure broader applicability of the results, future studies should strive for larger and more varied sample sizes.
2. Homogeneity of Participants: We concentrated on university students, potentially limiting the wider applicability of our findings. Future work should seek to incorporate a demographically broader participant cohort, with varying ages, cultural contexts, and life experiences to bolster the universality of the results.
3. Arbitrary Selection of Data Pruning Threshold: One potential limitation in our study lies in the arbitrary selection of the N/2 threshold for data pruning. While this threshold was chosen to balance reliability and precision, it may not be the optimal choice for all datasets and research objectives. To address this limitation and refine our approach, future studies should explore the selection of data pruning thresholds in a more systematic and data-driven manner. This could involve conducting sensitivity analyses with different threshold values to identify the thresholds that maximize model performance, particularly with respect to reliability and precision. Such investigations can contribute to a more evidence-based and robust data pruning process in the context of attachment style prediction through EEG data analysis.
4. Engineering Enhancements: Technologically, it would be advantageous for subsequent studies to investigate the creation of real-time EEG data processing systems to predict attachment styles. Such systems could prove invaluable in clinical settings for immediate interventions. Improving the model’s computational efficiency for use on devices with limited resources would also be beneficial, facilitating wider deployment and access.
5. Longitudinal Analysis: Incorporating a longitudinal study design could provide insights into the stability of attachment styles over time and the model’s predictive power across different life stages.
6. Interdisciplinary Approaches: Combining psychological theory with advanced machine learning techniques can be further explored. For example, integrating findings from neurobiology and psychodynamic theory might refine the model’s predictive capabilities.
7. Cross-Validation with Behavioral Data: To enhance the validity of our model, future studies could incorporate behavioral measures of attachment, providing a multimodal approach to validation and potentially capturing a more holistic picture of attachment patterns.

Addressing these limitations and incorporating the proposed future directions could improve the understanding and prediction of attachment styles. This progress may benefit psychological practices and contribute to more tailored therapeutic approaches.

### 4.7. Conclusion

In conclusion, our study offers a tangible example of how AI can be harnessed to predict complex psychological constructs with a high degree of accuracy, using EEG data. It underscores the significance of precision and careful model engineering when handling the complex signals typically found in psychological assessments. These two stages, comprising CatBoost-driven baseline predictions and iterative data refinement, form the cornerstone of our approach to improving attachment style prediction through EEG data analysis, with the use of ROCKET for feature extraction.

Our approach—balancing data inclusivity against the necessity for data pruning—reflects an ongoing trend in AI development towards more tailored, specialized, and efficient systems. By detailing the parameters and decision-making processes behind our model, we hope to add a useful perspective to the collective effort of applying AI in complex scientific fields. Our findings may inspire further exploration and cautious application of AI within the realms of neuroscience and psychology, potentially leading to new avenues for research and understanding in these interdisciplinary areas.
